# 6,7,8,9,10,11-Hexahydro-13*H*-azocino[2,1-*b*]quinazolin-13-one

**DOI:** 10.1107/S160053680902460X

**Published:** 2009-07-04

**Authors:** Rasul Ya. Okmanov, Zafir U. Samarov, Kambarali K. Turgunov, Bakhodir Tashkhodjaev, Khusniddin M. Shakhidoyatov

**Affiliations:** a S. Yunusov Institute of the Chemistry of Plant Substances, Academy of Sciences of Uzbekistan, Mirzo Ulugbek Str. 77, Tashkent 100170, Uzbekistan

## Abstract

The title compound, C_14_H_16_N_2_O, is a synthetic analogue of quinazolone alkaloids with pyrrilo, pyrido and azopino rings. The quinazolinic part of the mol­ecule is generally planar within 0.037 (3) Å; the eight-membered ring exhibits an inter­mediate conformation between the chair and boat forms as it is typical for cyclo­octene rings. An ethyl­ene group of the azopino ring is disordered over two positions with a refined occupancy ratio of 0.910 (7):0.090 (7). In the crystal, the H atoms of the aromatic rings form weak C—H⋯O and C—H⋯N hydrogen bonds. One C—H⋯O hydrogen bond leads to the formation of a one-dimensional chain. Another C—H⋯O and a C—H⋯N bond link these chains, generating a three-dimensional network.

## Related literature

For the synthesis of the title compound, see: Shakhidoyatov *et al.* (1976 ▶). For its physiological activity, see: Shakhidoyatov (1988 ▶). For crystal structures of pyrido-quinazolone and azopino-quinazolone, see: Tashkhodzhaev *et al.* (1995 ▶). For spectroscopic data and the chemical structures of pyrido-quinazoline and -quinazolone alkaloids, see: Turgunov *et al.* (1995 ▶). For cyclo­octene ring conformations, see: Barnes *et al.* (1992 ▶). For weak hydrogen bonds in alkaloids, see: Rajnikant *et al.* (2005 ▶).
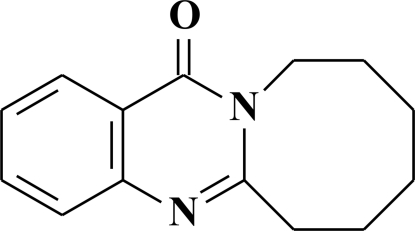


## Experimental

### 

#### Crystal data

C_14_H_16_N_2_O
*M* *_r_* = 228.29
Orthorhombic, 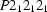

*a* = 9.5490 (19) Å
*b* = 10.584 (2) Å
*c* = 11.693 (2) Å
*V* = 1181.8 (4) Å^3^
*Z* = 4
Mo *K*α radiation
μ = 0.08 mm^−1^
*T* = 300 K
0.60 × 0.42 × 0.35 mm

#### Data collection

Stoe Stadi-4 four-circle diffractometer
Absorption correction: none
1229 measured reflections
1208 independent reflections
1061 reflections with *I* > 2σ(*I*)
3 standard reflections frequency: 60 min intensity decay: 3.9%

#### Refinement

*R*[*F* ^2^ > 2σ(*F* ^2^)] = 0.035
*wR*(*F* ^2^) = 0.079
*S* = 1.17
1208 reflections
174 parameters
H-atom parameters constrained
Δρ_max_ = 0.14 e Å^−3^
Δρ_min_ = −0.11 e Å^−3^

### 

Data collection: *STADI4* (Stoe & Cie, 1997 ▶); cell refinement: *STADI4*; data reduction: *X-RED* (Stoe & Cie, 1997 ▶); program(s) used to solve structure: *SHELXS97* (Sheldrick, 2008 ▶); program(s) used to refine structure: *SHELXL97* (Sheldrick, 2008 ▶); molecular graphics: *XP* (Bruker, 1998 ▶) and *Mercury* (Macrae *et al.*, 2006 ▶); software used to prepare material for publication: *SHELXL97*.

## Supplementary Material

Crystal structure: contains datablocks I, global. DOI: 10.1107/S160053680902460X/zl2205sup1.cif

Structure factors: contains datablocks I. DOI: 10.1107/S160053680902460X/zl2205Isup2.hkl

Additional supplementary materials:  crystallographic information; 3D view; checkCIF report

## Figures and Tables

**Table 1:** Hydrogen-bond geometry (Å, °)

*D*—H⋯*A*	*D*—H	H⋯*A*	*D*⋯*A*	*D*—H⋯*A*
C8—H8*A*⋯O1^i^	0.93	2.64	3.490 (3)	153
C9—H9*A*⋯N7^ii^	0.93	2.74	3.660 (3)	170
C10—H10*A*⋯O1^iii^	0.93	2.71	3.599 (3)	162

Symmetry codes: (i) 
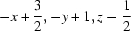
; (ii) 
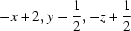
; (iii) 
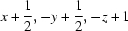
.

**Fig. 1.**
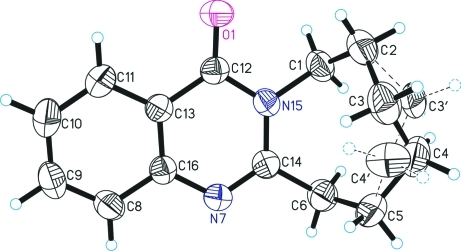
The molecular structure of the title compound, with 50% probability displacement ellipsoids (bonds of the minor disordered moiety are represented by dashed lines).

**Fig. 2.**
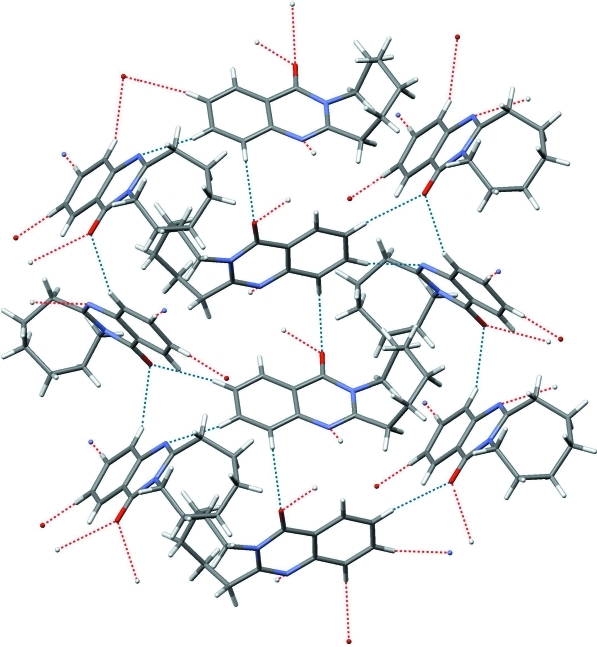
The H-bonding networks in the crystal of the title compound. Minor moiety disordered atoms are omitted for clarity.

**Fig. 3.**
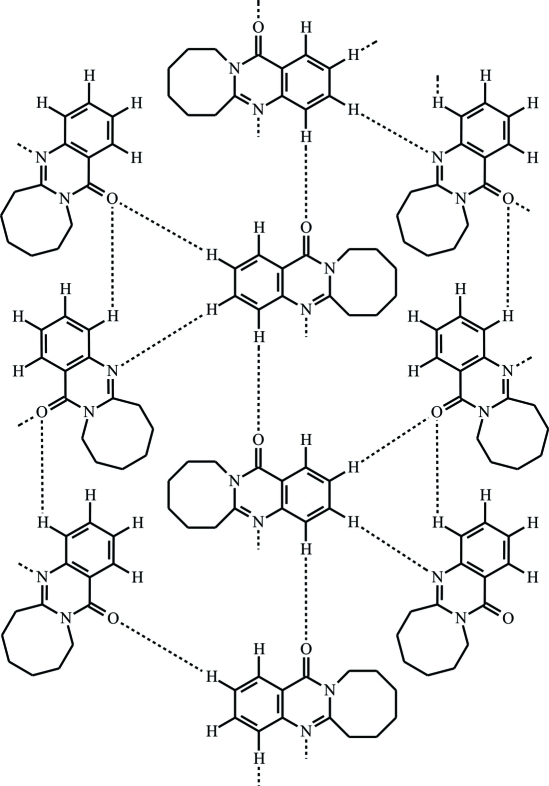
Schematic showing the weak hydrogen bonds (dashed lines).

## supplementary crystallographic information

### Comment

Tricyclic quinazolin-4-ones with polymethylenic fragments and their analogues are widely spread in plants and possess various physiological activities (Shakhidoyatov, 1988). With this in mind the title compound was synthesized (Shakhidoyatov *et al.*, 1976) and its crystal structure has been investigated by single crystal X-ray diffraction.

Figure 1 shows an ortep style plot of the molecular structure of the title compound. An ethylene group of the molecule is disordered over two positions (C3, C3', C4, C4'). Refinement of the structure yielded an occupancy ratio of the disordered atoms (*i.e.* two conformers) of 0.910 (7):0.090 (7).

The quinazoline part of the molecule is a generally flat within a standard deviation of ±0.037 Å. The electronic system of the N7—C14—N15—C12 fragment of the pyrymidinic ring is delocalized as reflected by the bond lengths. The length of the formal double bond N7═C14 and the single bond C14—N15 in the structure of the title compound are 1.297 (3) and 1.383 (3) Å, respectively, which is in agreement with the range observed in crystals of pyrrilo, pyrido, and azopino quinazolones (Turgunov *et al.*, 1995; Tashkhodzhaev *et al.*, 1995). The length of the C═O bond (1.227 (3) Å) is also compareable to those observed in above mentioned analogues. The eight-membered ring has taken on an intermediate form between a chair and boat conformation typical for cycloectene rings (Barnes *et al.*, 1992).

In the crystal structure of the title compound weak intermolecular C-H···X hydrogen bonds are observed as it is often the case in alkaloids (Rajnikant *et al.*, 2005). The hydrogen bond C8–H8A···O1^i^ leads to the formation of a one dimensional chain. Another C–H···O and a C–H···N bond (C10–H10A···O1^iii^ and a C9–H9A···N7^ii^) link these chains to generate a three-dimensional network (Fig. 2 and 3; for numerical values and symmetry operators see Table 1).

### Experimental

The title compound was synthesized on the basis of a well–known method (Shakhidoyatov, *et al.*, 1976). Powder of title compound was dissolved in hot aqueous ethanol and from the solution yellow prismatic crystals were obtained during slow evaporation in a thermostat at a temperature of 313 K.

### Refinement

In the absence of anomalous scatteres and using molybdenum radiation Friedel pairs were merged prior to refinement. The C3 and C4 atoms of the molecule are disordered over two positions (C3, C3', C4, C4'). Refinement of the structure by using a free variable for the occupancy led to a ratio for the disordered atoms of 0.910 (7):0.090 (7). The bond lengths of the disordfered hexamethylenic fragment were restrained to be the same within a standard deviation of 0.02 Å.

The H atoms bonded to C atoms were placed geometrically (with C—H distances of 0.97 Å for CH_2_ and 0.93 Å for C_ar_) and included in the refinement with a riding motion approximation with U_iso_ = 1.2U_eq_(C) [U_iso_ = 1.5U_eq_(C) for methyl H atoms].

### Crystal data

**Table:**

C_14_H_16_N_2_O	*D*_x_ = 1.283 Mg m^−^^3^
*M**_r_* = 228.29	Melting point: 391(3) K
Orthorhombic, *P*2_1_2_1_2_1_	Mo *K*α radiation, λ = 0.71073 Å
Hall symbol: P 2ac 2ab	Cell parameters from 12 reflections
*a* = 9.5490 (19) Å	θ = 10–15°
*b* = 10.584 (2) Å	µ = 0.08 mm^−^^1^
*c* = 11.693 (2) Å	*T* = 300 K
*V* = 1181.8 (4) Å^3^	Prizmatic, yellow
*Z* = 4	0.60 × 0.42 × 0.35 mm
*F*(000) = 488	

### Data collection

**Table:**

Stoe Stadi-4 four-circle diffractometer	*R*_int_ = 0.0000
Radiation source: fine-focus sealed tube	θ_max_ = 25.0°, θ_min_ = 2.6°
graphite	*h* = 0→11
ω/2θ scans	*k* = 0→12
1229 measured reflections	*l* = 0→13
1208 independent reflections	3 standard reflections every 60 min
1061 reflections with *I* > 2σ(*I*)	intensity decay: 3.9%

### Refinement

**Table:**

Refinement on *F*^2^	Secondary atom site location: difference Fourier map
Least-squares matrix: full	Hydrogen site location: inferred from neighbouring sites
*R*[*F*^2^ > 2σ(*F*^2^)] = 0.035	H-atom parameters constrained
*wR*(*F*^2^) = 0.079	*w* = 1/[σ^2^(*F*_o_^2^) + (0.0252*P*)^2^ + 0.261*P*] where *P* = (*F*_o_^2^ + 2*F*_c_^2^)/3
*S* = 1.17	(Δ/σ)_max_ < 0.001
1208 reflections	Δρ_max_ = 0.14 e Å^−^^3^
174 parameters	Δρ_min_ = −0.10 e Å^−^^3^
0 restraints	Extinction correction: *SHELXL97* (Sheldrick, 2008), Fc^*^=kFc[1+0.001xFc^2^λ^3^/sin(2θ)]^-1/4^
Primary atom site location: structure-invariant direct methods	Extinction coefficient: 0.040 (3)

### Special details

**Table:**

Experimental. Scan width (omega) = 1.56 - 1.68, scan ratio 2theta:omega = 1.00 I(Net) and sigma(I) calculated according to Blessing, (1987).
Geometry. All e.s.d.'s (except the e.s.d. in the dihedral angle between two l.s. planes) are estimated using the full covariance matrix. The cell e.s.d.'s are taken into account individually in the estimation of e.s.d.'s in distances, angles and torsion angles; correlations between e.s.d.'s in cell parameters are only used when they are defined by crystal symmetry. An approximate (isotropic) treatment of cell e.s.d.'s is used for estimating e.s.d.'s involving l.s. planes.
Refinement. Refinement of *F*^2^ against ALL reflections. The weighted *R*-factor *wR* and goodness of fit *S* are based on *F*^2^, conventional *R*-factors *R* are based on *F*, with *F* set to zero for negative *F*^2^. The threshold expression of *F*^2^ > σ(*F*^2^) is used only for calculating *R*-factors(gt) *etc*. and is not relevant to the choice of reflections for refinement. *R*-factors based on *F*^2^ are statistically about twice as large as those based on *F*, and *R*- factors based on ALL data will be even larger.

### Fractional atomic coordinates and isotropic or equivalent isotropic displacement parameters (Å^2^)

**Table:**

	*x*	*y*	*z*	*U*_iso_*/*U*_eq_	Occ. (<1)
O1	0.48619 (19)	0.50852 (16)	0.48326 (16)	0.0541 (5)	
C1	0.4342 (3)	0.7278 (3)	0.3757 (2)	0.0501 (7)	
H1A	0.3555	0.6703	0.3823	0.060*	
H1B	0.4167	0.7825	0.3107	0.060*	
C2	0.4418 (3)	0.8074 (3)	0.4824 (3)	0.0626 (8)	
H2A	0.4389	0.7519	0.5483	0.075*	
H2B	0.3593	0.8608	0.4855	0.075*	
C3	0.5710 (4)	0.8909 (3)	0.4918 (3)	0.0625 (11)	0.910 (7)
H3A	0.6529	0.8373	0.4990	0.075*	0.910 (7)
H3B	0.5639	0.9409	0.5611	0.075*	0.910 (7)
C4	0.5920 (5)	0.9800 (3)	0.3902 (4)	0.0672 (12)	0.910 (7)
H4A	0.5024	0.9930	0.3530	0.081*	0.910 (7)
H4B	0.6234	1.0612	0.4188	0.081*	0.910 (7)
C3'	0.492 (4)	0.938 (3)	0.437 (4)	0.076 (16)	0.090 (7)
H3C	0.4513	0.9551	0.3629	0.091*	0.090 (7)
H3D	0.4626	1.0046	0.4895	0.091*	0.090 (7)
C4'	0.654 (3)	0.934 (5)	0.429 (3)	0.057 (11)	0.090 (7)
H4C	0.6935	1.0078	0.4666	0.068*	0.090 (7)
H4D	0.6892	0.8593	0.4667	0.068*	0.090 (7)
C5	0.6969 (3)	0.9334 (3)	0.3012 (3)	0.0659 (9)	
H5A	0.7816	0.9085	0.3411	0.079*	
H5B	0.7209	1.0042	0.2523	0.079*	
C6	0.6522 (3)	0.8230 (3)	0.2240 (2)	0.0556 (8)	
H6A	0.5559	0.8366	0.2007	0.067*	
H6B	0.7095	0.8247	0.1555	0.067*	
N7	0.7692 (2)	0.62498 (19)	0.24573 (17)	0.0431 (5)	
C8	0.8961 (3)	0.4294 (3)	0.2602 (2)	0.0504 (7)	
H8A	0.9545	0.4558	0.2012	0.061*	
C9	0.9186 (3)	0.3150 (3)	0.3122 (3)	0.0563 (8)	
H9A	0.9923	0.2642	0.2880	0.068*	
C10	0.8327 (3)	0.2743 (3)	0.4005 (3)	0.0562 (8)	
H10A	0.8505	0.1974	0.4361	0.067*	
C11	0.7218 (3)	0.3469 (2)	0.4355 (2)	0.0485 (7)	
H11A	0.6638	0.3192	0.4943	0.058*	
C12	0.5754 (3)	0.5395 (2)	0.4132 (2)	0.0377 (6)	
C13	0.6967 (2)	0.4634 (2)	0.38186 (19)	0.0366 (6)	
C14	0.6629 (3)	0.6936 (2)	0.2763 (2)	0.0400 (6)	
N15	0.5625 (2)	0.65339 (18)	0.35342 (16)	0.0385 (5)	
C16	0.7848 (2)	0.5065 (2)	0.2960 (2)	0.0379 (6)	

### Atomic displacement parameters (Å^2^)

**Table:**

	*U*^11^	*U*^22^	*U*^33^	*U*^12^	*U*^13^	*U*^23^
O1	0.0525 (11)	0.0515 (11)	0.0582 (11)	−0.0064 (10)	0.0185 (10)	0.0010 (10)
C1	0.0372 (13)	0.0490 (15)	0.0642 (17)	0.0079 (12)	0.0023 (13)	−0.0053 (14)
C2	0.0607 (18)	0.0529 (16)	0.0743 (19)	0.0055 (16)	0.0161 (17)	−0.0140 (16)
C3	0.063 (2)	0.058 (2)	0.067 (2)	0.006 (2)	−0.007 (2)	−0.0217 (19)
C4	0.069 (3)	0.0387 (19)	0.094 (3)	0.002 (2)	−0.002 (3)	−0.007 (2)
C3'	0.11 (4)	0.035 (18)	0.08 (3)	0.01 (2)	0.05 (3)	−0.010 (19)
C4'	0.04 (2)	0.08 (3)	0.04 (2)	0.00 (2)	−0.016 (17)	0.022 (19)
C5	0.0635 (19)	0.0414 (15)	0.093 (2)	0.0014 (15)	0.0113 (18)	0.0164 (17)
C6	0.0584 (17)	0.0541 (17)	0.0543 (16)	0.0136 (15)	0.0060 (14)	0.0176 (14)
N7	0.0404 (11)	0.0434 (12)	0.0456 (12)	0.0009 (10)	0.0059 (10)	0.0035 (10)
C8	0.0415 (15)	0.0526 (16)	0.0572 (17)	0.0040 (13)	0.0082 (14)	−0.0044 (15)
C9	0.0487 (16)	0.0490 (16)	0.0713 (19)	0.0148 (15)	−0.0032 (15)	−0.0076 (15)
C10	0.0618 (17)	0.0392 (14)	0.0674 (18)	0.0080 (14)	−0.0075 (17)	0.0010 (15)
C11	0.0537 (16)	0.0396 (14)	0.0523 (16)	−0.0029 (14)	−0.0010 (13)	0.0034 (12)
C12	0.0398 (13)	0.0368 (13)	0.0365 (12)	−0.0059 (11)	0.0001 (13)	−0.0044 (11)
C13	0.0368 (13)	0.0353 (12)	0.0377 (13)	−0.0032 (11)	−0.0011 (11)	−0.0058 (10)
C14	0.0418 (14)	0.0417 (14)	0.0366 (13)	0.0020 (12)	−0.0008 (12)	0.0004 (12)
N15	0.0362 (11)	0.0390 (11)	0.0401 (10)	0.0020 (10)	0.0014 (10)	−0.0052 (9)
C16	0.0382 (14)	0.0363 (12)	0.0392 (12)	−0.0005 (12)	−0.0027 (11)	−0.0030 (11)

### Geometric parameters (Å, °)

**Table:**

O1—C12	1.227 (3)	C5—C6	1.537 (4)
C1—N15	1.480 (3)	C5—H5A	0.9700
C1—C2	1.507 (4)	C5—H5B	0.9700
C1—H1A	0.9700	C6—C14	1.503 (4)
C1—H1B	0.9700	C6—H6A	0.9700
C2—C3	1.521 (4)	C6—H6B	0.9700
C2—C3'	1.56 (3)	N7—C14	1.298 (3)
C2—H2A	0.9700	N7—C16	1.393 (3)
C2—H2B	0.9700	C8—C9	1.371 (4)
C3—C4	1.530 (6)	C8—C16	1.404 (3)
C3—H3A	0.9700	C8—H8A	0.9300
C3—H3B	0.9700	C9—C10	1.387 (4)
C4—C5	1.526 (5)	C9—H9A	0.9300
C4—H4A	0.9700	C10—C11	1.371 (4)
C4—H4B	0.9700	C10—H10A	0.9300
C3'—C4'	1.55 (3)	C11—C13	1.404 (3)
C3'—H3C	0.9700	C11—H11A	0.9300
C3'—H3D	0.9700	C12—N15	1.398 (3)
C4'—C5	1.55 (3)	C12—C13	1.458 (3)
C4'—H4C	0.9700	C13—C16	1.387 (3)
C4'—H4D	0.9700	C14—N15	1.383 (3)
			
N15—C1—C2	113.8 (2)	C4—C5—H5A	107.9
N15—C1—H1A	108.8	C6—C5—H5A	107.9
C2—C1—H1A	108.8	C4'—C5—H5A	76.1
N15—C1—H1B	108.8	C4—C5—H5B	107.9
C2—C1—H1B	108.8	C6—C5—H5B	107.9
H1A—C1—H1B	107.7	C4'—C5—H5B	128.7
C1—C2—C3	115.1 (2)	H5A—C5—H5B	107.2
C1—C2—C3'	103.5 (16)	C14—C6—C5	115.8 (2)
C1—C2—H2A	108.5	C14—C6—H6A	108.3
C3—C2—H2A	108.5	C5—C6—H6A	108.3
C3'—C2—H2A	144.7	C14—C6—H6B	108.3
C1—C2—H2B	108.5	C5—C6—H6B	108.3
C3—C2—H2B	108.5	H6A—C6—H6B	107.4
C3'—C2—H2B	75.1	C14—N7—C16	118.1 (2)
H2A—C2—H2B	107.5	C9—C8—C16	119.9 (3)
C2—C3—C4	114.1 (4)	C9—C8—H8A	120.0
C2—C3—H3A	108.7	C16—C8—H8A	120.0
C4—C3—H3A	108.7	C8—C9—C10	120.8 (3)
C2—C3—H3B	108.7	C8—C9—H9A	119.6
C4—C3—H3B	108.7	C10—C9—H9A	119.6
H3A—C3—H3B	107.6	C11—C10—C9	120.3 (3)
C5—C4—C3	114.6 (4)	C11—C10—H10A	119.8
C5—C4—H4A	108.6	C9—C10—H10A	119.8
C3—C4—H4A	108.6	C10—C11—C13	119.4 (3)
C5—C4—H4B	108.6	C10—C11—H11A	120.3
C3—C4—H4B	108.6	C13—C11—H11A	120.3
H4A—C4—H4B	107.6	O1—C12—N15	120.2 (2)
C4'—C3'—C2	108 (4)	O1—C12—C13	124.9 (2)
C4'—C3'—H3C	110.2	N15—C12—C13	114.9 (2)
C2—C3'—H3C	110.2	C16—C13—C11	120.6 (2)
C4'—C3'—H3D	110.2	C16—C13—C12	118.8 (2)
C2—C3'—H3D	110.2	C11—C13—C12	120.6 (2)
H3C—C3'—H3D	108.5	N7—C14—N15	123.3 (2)
C5—C4'—C3'	109 (3)	N7—C14—C6	116.8 (2)
C5—C4'—H4C	109.9	N15—C14—C6	119.9 (2)
C3'—C4'—H4C	109.9	C14—N15—C12	122.0 (2)
C5—C4'—H4D	109.9	C14—N15—C1	121.7 (2)
C3'—C4'—H4D	109.9	C12—N15—C1	116.3 (2)
H4C—C4'—H4D	108.3	C13—C16—N7	122.4 (2)
C4—C5—C6	117.7 (3)	C13—C16—C8	119.0 (2)
C6—C5—C4'	119.9 (16)	N7—C16—C8	118.6 (2)

### Hydrogen-bond geometry (Å, °)

**Table:**

*D*—H···*A*	*D*—H	H···*A*	*D*···*A*	*D*—H···*A*
C8—H8A···O1^i^	0.93	2.64	3.490 (3)	153
C9—H9A···N7^ii^	0.93	2.74	3.660 (3)	170
C10—H10A···O1^iii^	0.93	2.71	3.599 (3)	162

Symmetry codes: (i) −*x*+3/2, −*y*+1, *z*−1/2; (ii) −*x*+2, *y*−1/2, −*z*+1/2; (iii) *x*+1/2, −*y*+1/2, −*z*+1.
